# Perioperative safety assessment of patients undergoing unilateral or bilateral hip replacements

**DOI:** 10.3389/fsurg.2023.944311

**Published:** 2023-01-26

**Authors:** Zhenzhong Zhou, Gaorui Cai, Shanyou Yuan, Lixia Song, Ping Qian, Xueming Wang, Xianjia Ning, Jinghua Wang, Wenxue Jiang

**Affiliations:** ^1^ Department of Orthopedics, The Third People's Hospital of Shenzhen, Guangdong, China; ^2^ Center of Clinical Epidemiology, The Third People's Hospital of Shenzhen, Guangdong, China

**Keywords:** safety assessment, perioperative, total hip replacements, lateral, blood loss

## Abstract

**Introduction:**

Due to the aging of the world population and the increase of obesity rate, it is expected that the number of joint replacement surgery will continue to increase in the next few years. This study evaluated the safety differences between unilateral and bilateral hip replacement surgeries.

**Methods:**

The data for patients who underwent hip arthroplasty in 2021 and 2022 were examined. The data set included 68 patients who were grouped according to the type of hip replacement needed, sex, age, and body mass index. Total blood loss and operative time were the safety-related indicators used to compare the groups.

**Results:**

Regardless of whether the unilateral replacement group was compared with the overall bilateral replacement group or separately with the staged and simultaneous bilateral replacement groups, simultaneous bilateral replacement surgeries were equally safe as the other types of hip replacements. The total blood loss and operative time for the simultaneous bilateral replacement group were not significantly different from those in the unilateral and staged bilateral replacement groups. For overweight patients, the operative time for simultaneous bilateral replacements was significantly shorter than that for unilateral replacements.

**Conclusions:**

These findings suggest that for patients requiring bilateral hip replacements, the blood loss risk for patients undergoing simultaneous bilateral hip replacements was similar to that for patients undergoing either unilateral or staged bilateral hip replacements. Thus, simultaneous bilateral total hip replacement (THR) are safe and should be considered for candidate patients.

## Introduction

Total hip replacement (THR) and total knee replacement (TKR) are two of the most common orthopedic surgeries performed in the United Kingdom (1). According to the Organization for Economic Co-operation and Development, 283 hip replacements and 190 knee replacements, per 100,000 people, were performed in Germany in 2013 (2). Moreover, data extrapolations have shown that German statutory health insurances spent approximately 1.4–1.6 billion euros per year on THRs between 2003 and 2009 (3). Between 2011 and 2019, the number of TKA cases in China increased rapidly, from 53,880 to 374,833, a 5.9-fold increase, and TKR also showed a significant increase (4).

Previous studies have shown that, in many industrialized countries, the numbers of hip and knee replacements are rising. Furthermore, due to aging populations and increasing obesity rates, further increases in the number of joint replacement surgeries can be expected (5); the frequency of simultaneous bilateral procedures has also been increasing (6). Some studies have noted that although THR is a successful procedure for many patients, the procedure also has the potential for post-operative complications, including bleeding, wound dehiscence, infection, and dislocation (7). However, whether there are differences between the safety of unilateral and simultaneous bilateral hip replacement surgeries remains controversial. Therefore, this study evaluated whether there are differences in safety parameters associated with unilateral and bilateral hip replacements.

## Method

### Study design

This hospital-based, single-center, prospective study recruited consecutive patients undergoing hip replacement surgeries in 2021 and 2022 at The Third People's Hospital of Shenzhen. In this study, those patients who underwent the hip replacement both for bilateral and for unilateral hip replacement were included and assessed.

All patients were grouped according to side and operation period. The participants were divided into two groups according to whether they underwent unilateral or bilateral hip replacement operations. Those who underwent bilateral hip replacement operations were further divided into the simultaneous and staged bilateral groups, depending on whether the hips were replaced in a single surgery or during two separate operations, respectively. Subgroups were also established based on patient sex, age, and body mass index (BMI).

### Patient inclusion and exclusion criteria

Patients were included if they were ≥18 years old and needed hip replacement(s) due to femoral neck fractures or femoral head necrosis caused by factors such as osteoarthritis and alcoholic osteonecrosis of the femoral head. All patients had elective surgery with no history of trauma.

The exclusion criteria included a previous history of abnormal nutritional and coagulation functions or complications resulting from infections or tumors at the surgical site.

### Surgical process

**Figure 1:**
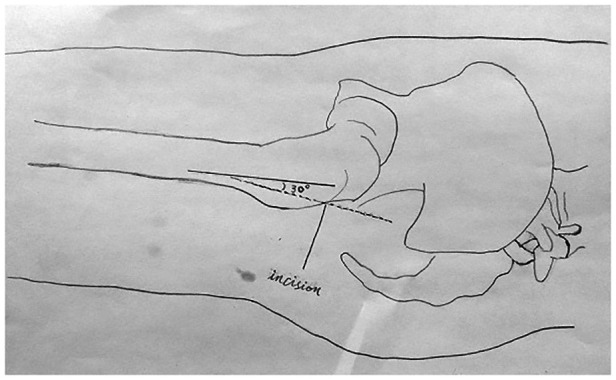
The modified hip incision. The modified hip incision, 5 cm below the apex of the greater trochanter, was made at a 30 degree angle to the femur, and the oblique incision was made upward about 8 to 12 cm.

**Figure 2:**
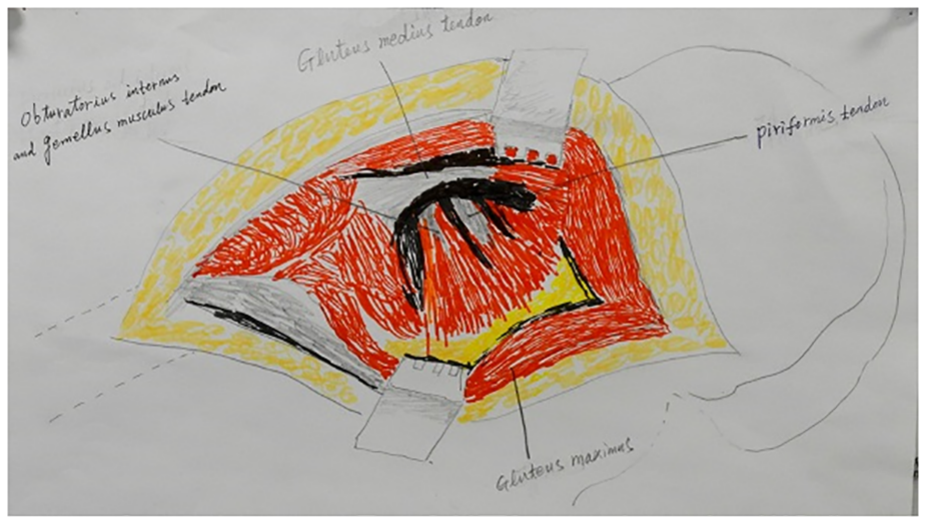
Exposing the femoral neck. The piriformis and obturator internus muscles and the superior and inferior gemellus tendons were severed to expose the femoral neck.

For each involved hip, a modified posterolateral hip incision was made such that the posterior edge of the greater trochanter was oblique to the posterior straight incision and the proximal axis of the femoral shaft was at 30° to the incision (Figure 1). The upper edge of the incision was to the vertical line of the anterior superior iliac spine and the lower edge was 5 cm below the greater trochanter. The fasciae of the gluteus maximus, gluteus medius, and external rotators were incised along the incision. A Hohman retractor was inserted under the gluteus medius and retracted medially to expose the piriformis and gluteus minimus. The insertion of the piriformis, superior and inferior gemellus and joint capsule were dissected from the piriform fossa (Figure 2). The capsule was then opened using a T-shaped incision on the acetabular side, and the hip joint was flexed and internally rotated to yield a posterior dislocation. Thereafter, oblique amputation of the femoral neck was performed 1 cm above the lesser trochanter and the femoral head was removed. The acetabular labrum was resected, the acetabulum was ground to the subchondral bone, and the acetabular prosthesis was installed. The medullary cavity was reamed on the femoral side, followed by expansion of the medullary cavity, washing and removal of bone fragments, insertion of an artificial femoral stem trial mold, and installation of the prosthesis after confirming satisfactory C-arm fluoroscopy results. The wound was thoroughly washed and the bleeding stopped. A negative pressure drainage tube was inserted into the wound and the piriformis muscle was sutured to the tendon insertion of the gluteus medius; the incision was closed layer by layer.

### Information collected

Demographic information including age, sex, and educational level, were collected. Personal medical histories including histories of hypertension, diabetes, coronary heart disease, and smoking and alcohol consumption statuses were collected using pre-designed questionnaire.

Anthropometric measurements including systolic and diastolic blood pressures and BMI were recorded during physical examinations. Overweight was defined as a BMI of 24.0–28.0 kg/m^2^ (8), and obesity was defined as a BMI ≥ 28.0 kg/m^2^ (8).

### Safety evaluations

Total blood losses and operative times were used as the tracked safety indicators. Complications, such as poor incision healing, intestinal obstruction, deep venous thrombosis, and pulmonary infection, were evaluated during the perioperative period.

Intraoperative blood loss, blood transfusions, and wound drainage volumes within 24 h were tracked in conjunction with other perioperative data. Total blood loss was calculated according to the Gross equation (9), and the theoretical total blood loss was calculated using the serum hematocrit (HCT). Theoretical total blood loss = PBV × (preoperative HCT—postoperative HCT)/average HCT, where PBV = K1 × H3 + K2 × W + K3; PBV is the preoperative blood volume, H is the height (m) of the patient and W is the individual's weight (kg). For male patients, K1 = 0.3669, K2 = 0.03219, and K3 = 0.6041; for female patients, K1 = 0.3561, K2 = 0.03308, and K3 = 0.1833 (10). To calculate hidden blood loss, the following equations were used: apparent blood loss = intraoperative blood loss + postoperative blood loss; hidden blood loss = total blood loss—apparent blood loss (11); total blood loss = theoretical total blood loss + total blood transfusion volume.

### Statistical analysis

Patients were analyzed on two levels. First, patients were grouped into two groups: unilateral and bilateral group, depending on the number of damaged hip joints (defined as Level 1). Second, patients were grouped into three groups: unilateral group, staged bilateral replacement group, and simultaneous bilateral replacement group (defined as Level 2).

All continuous variables (including age; BMI; levels of C-reactive protein (CRP), procalcitonin (PCT), alanine transaminase (ALT), aspartate transaminase (APT); estimated glomerular filtration rate (eGFR), red blood cell (RBC) count, HCT, platelet count, neutrophil count, total blood loss, operative time, preoperative HCT, and postoperative HCT) are presented as means and standard deviations for the Level 1 analyses. All categorical variables (including case; sex; age group; education level; presence of hypertension, diabetes, coronary heart disease, and HIV; BMI group; and complications) are presented as numbers and percentages for the Level 2 analyses. Between group comparisons were made using Student's t-test or the Mann–Whitney U-test for total blood losses and operative times and using the chi-squared test for Level 1 and 2 analyses, respectively. Moreover, the subgroup safety analyses were assessed using sex, age group, and BMI group. All significance tests were two-sided, and *P* values < 0.05 were considered significant. SPSS version 19.0 for Windows (SPSS, Chicago, IL, USA) was used for analyses.

## Results

### Patient demographic characteristics

The study population consisted of 68 patients, including 41 (60.3%) men and 27 (39.7%) women, who underwent hip replacements during the study period; 50 (73.5%) underwent unilateral replacements and 18 (26.5%) underwent bilateral replacements. Additionally, 48.5% of the patients were in the <45 years group and >60% had attained secondary education. The average age of the patients was 46.53 (12.64) years, with the patients in the unilateral replacement group being older than those in the bilateral replacement group (49.90 years vs. 37.17 years, *P* < 0.001). Normal, overweight, and obese patients accounted for 70.6%, 19.1%, and 10.3% of the included patients, respectively. There were no significant differences in the gender group; education levels; previous histories of hypertension, diabetes, coronary heart disease, HIV; levels of CRP, PCT, ALT, and AST; eGFR, RBC count, HCT, platelet counts, and neutrophil counts between the groups (all, *P* > 0.05) (Table 1).

**Table 1:** Baseline characteristic in all patients both in unilateral and bilateral. .

Factors	Total	Unilateral	Bilateral	*P*
Gender, *n* (%):				0.934
Men	41 (60.3)	30 (60.0)	11 (61.1)	
Women	27 (39.7)	20 (40.0)	7 (38.9)	
Age, mean (SD), years	46.53 (12.64)	49.90 (12.27)	37.17 (8.37)	<0.001
Age group, *n* (%):				0.001
<45 years	33 (48.5)	18 (36.0)	15 (83.3)	
45 years∼	29 (42.6)	26 (52.0)	3 (16.7)	
≥65 years∼	6 (8.8)	6 (12.0)	0	
Education, *n* (%):				0.337
Primary education	19 (27.9)	16 (32.0)	3 (16.7)	
Secondary education	42 (61.8)	30 (60.0)	12 (66.6)	
University education	7 (10.3)	4 (8.0)	3 (16.7)	
Hypertension, *n* (%):	5 (7.4)	5 (10.0)	0	0.315
Diabetes, *n* (%):	1 (1.5)	1 (2.0)	0	1.000
Coronary heart disease, *n* (%)	1 (1.5)	1 (2.0)	0	1.000
HIV disease, *n* (%)	20 (29.4)	13 (26.0)	7 (38.9)	0.303
BMI, means (SD), kg/m^2^	21.92 (5.26)	23.18 (3.56)	18.39 (7.40)	0.019
BMI groups, *n* (%)				0.090
Normal weight	48 (70.6)	33 (66.0)	15 (83.3)	
Overweight	13 (19.1)	10 (20.0)	3 (16.7)	
Obesity	7 (10.3)	7 (14.0)	0	
CRP, mg/L, means (SD)	12.44 (19.80)	11.54 (16.62)	14.42 (25.90)	0.624
PCT, mg/L, means (SD)	0.13 (0.16)	0.10 (0.09)	0.23 (0.24)	0.083
ALT, mg/L, means (SD)	47.16 (48.63)	19.90 (15.39)	14.23 (9.09)	0.158
AST, mg/L, means (SD)	12.44 (19.80)	21.05 (8.89)	22.41 (24.27)	0.738
eGFR, mg/L, means (SD)	0.13 (0.16)	101.95 (22.19)	113.58 (30.52)	0.093
RBC, 10^9^/L, means (SD)	18.44 (14.19)	4.30 (0.70)	4.42 (0.66)	0.510
HCT, 10^9^/L, means (SD)	38.12 (7.47)	38.25 (7.05)	37.65 (8.97)	0.765
Platelet, 10^9^/L, means (SD)	105.12 (25.04)	237.94 (88.44)	245.72 (64.68)	0.734
NEUT, 10^9^/L, means (SD)	21.40 (14.34)	54.24 (14.97)	56.79 (13.91)	0.529

### Level 1 safety comparison

The safety evaluation examined only total blood losses and operative times because none of the patients demonstrated poor incision healing, deep venous thrombosis, or pulmonary infection; only one patient (unilateral replacement group) developed an intestinal obstruction.

There were no significant safety differences between the unilateral and bilateral groups (Table 2). In the subgroup analysis based on sex (men vs. women), age (<45 years vs. ≥45 years), and BMI (normal weight, overweight, and obesity), the operative time was shorter for the bilateral group than for the unilateral group among patients who were overweight (*P* < 0.001). There were no significant differences in the total blood losses or operative times between the unilateral and bilateral groups across the other subgroups.

**Table 2:** The security evaluation among patients in unilateral and bilateral.

Factors	Unilateral	Bilateral	*P*
Total:			* *
TLB, ml, means (SE)	293.84 (129.48)	193.32 (48.15)	0.377
Operation time, minutes, means (SE)	145.50 (21.82)	129.44 (9.28)	0.442
Sex:			
Men:			
TLB, ml, means (SE)	368.20 (187.50)	154.67 (52.67)	0.293
Operation time, minutes, means (SE)	164.42 (31.31)	133.82 (12.62)	0.287
Women:			
TLB, ml, means (SE)	145.13 (98.45)	254.51 (92.49)	0.576
Operation time, minutes, means (SE)	107.67 (10.68)	122.50 (13.45)	0.397
Age groups:			
<45 years:			
TLB, ml, means (SE)	327.49 (149.62)	193.50 (64.37)	0.419
Operation time, minutes, means (SE)	144.33 (23.85)	155.18 (13.05)	0.739
≥45 years:			
TLB, ml, means (SE)	277.02 (183.58)	193.12 (73.56)	0.611
Operation time, minutes, means (SE)	146.08 (31.21)	100.14 (11.03)	0.187
BMI groups:			
Normal weight:			
TLB, ml, means (SE)	196.28 (79.40)	185.68 (63.66)	0.932
Operation time, minutes, means (SE)	122.62 (16.69)	123.70 (10.63)	0.960
Overweight:			
TLB, ml, means (SE)	1112.43 (1110.32)	145.44 (75.79)	0.544
Operation time, minutes, means (SE)	357.50 (4.50)	135.08 (26.83)	<0.001
Obesity:			
TLB, ml, means (SE)	170.89 (169.36)	322.32 (114.05)	0.485
Operation time, minutes, means (SE)	103.33 (4.98)	155.00 (21.78)	0.173

### Level 2 safety comparison

Similar trends were found in the Level 2 analysis (Table 3). Overall, there were no significant differences in total blood losses or operative times between the unilateral, simultaneous bilateral, and staged bilateral groups. However, the operative time for the unilateral replacement group was significantly longer for patients in the overweight group than for those in either of the bilateral replacement groups (*P* < 0.05). Significant differences in total blood losses or operative times were not observed between the three surgical groups when the other subgroups were examined: sex (men vs. women), age (<45 years vs. ≥45 years), and BMI (normal weight vs. obesity).

**Table 3:** The security evaluation among patients in unilateral, bilateral two times, and bilateral one time.

Factors	Unilateral	Bilateral two times	Bilateral one times	*P*
Total:				* *
TLB, ml, means (SD)	293.84 (129.48)	220.35 (85.98)	176.24 (57.57)	0.626
Operation time, minutes, means (SD)	145.50 (21.82)	140.46 (82.95)	122.47 (10.75)	0.505
Sex:				
Men:				
TLB, ml, means (SD)	368.20 (187.50)	156.04 (68.53)	153.78 (75.89)	0.327
Operation time, minutes, means (SD)	164.42 (31.31)	145.53 (22.16)	126.17 (15.22)	0.454
Women:				
TLB, ml, means (SD)	145.13 (98.45)	327.54 (201.69)	210.69 (90.42)	0.696
Operation time, minutes, means (SD)	107.67 (10.68)	132.00 (27.40)	116.80 (15.54)	0.735
Age groups:				
<45 years:				
TLB, ml, means (SD)	327.49 (149.62)	85.18 (49.19)	263.90 (99.29)	0.289
Operation time, minutes, means (SD)	144.33 (23.85)	168.62 (25.11)	146.45 (14.29)	0.662
≥45 years:				
TLB, ml, means (SD)	277.02 (183.58)	380.11 (170.15)	78.85 (43.89)	0.221
Operation time, minutes, means (SD)	146.08 (31.21)	107.18 (18.45)	95.83 (14.07)	0.220
BMI groups:				
Normal weight:				
TLB, ml, means (SD)	196.28 (79.40)	196.64 (107.50)	177.79 (79.36)	0.985
Operation time, minutes, means (SD)	122.62 (16.69)	135.22 (19.61)	115.40 (11.72)	0.641
Overweight:				
TLB, ml, means (SD)	1112.43 (1110.32)	194.80 (192.88)	128.99 (85.42)	0.107
Operation time, minutes, means (SD)	357.50 (4.50)	149.33 (68.42)	130.33 (30.30)	0.029
Obesity:				
TLB, ml, means (SD)	170.89 (169.36)	388.16 (193.48)	272.93 (157.84)	0.712
Operation time, minutes, means (SD)	103.33 (4.98)	163.00 (41.36)	149.00 (27.89)	0.398

## Discussion

The purpose of this study was to evaluate the differences in safety parameters between patients undergoing unilateral or bilateral hip replacements. The findings indicated that regardless of whether the unilateral group was compared with the bilateral group, overall, or with the staged and simultaneous bilateral replacement groups, the simultaneous bilateral hip replacement group was determined to be safe. Total blood losses and operative times in the staged bilateral replacement group were similar to those in the unilateral or simultaneous bilateral replacement groups. The operative time was also shorter in the bilateral group than in the unilateral group for overweight patients and there were no significant differences between the unilateral and bilateral groups relative to total blood losses or operative times for the other subgroups (sex, age, and BMI).

A previous study showed that simultaneous bilateral THR is a safe and effective option for patients with significant arthritic disease of both hips (12), and the overall risk of complications following bilateral THR is similar to that seen following unilateral procedures (13). Similarly, meta-analyses have not shown increased risks of major complications for simultaneous bilateral THRs compared with unilateral THRs (14, 15), consistent with our findings. However, one study showed that simultaneous THR surgery is associated with an elevated risk of blood transfusion (16); another showed that simultaneous bilateral THR had a lower risk of major systemic complications, less deep venous thrombosis risk, and shorter operative times than staged bilateral THRs (17). Such incongruities may be explained by differences in population characteristics and technology levels. In addition, a previous study including 2036 patients undergoing bilateral simultaneous total hip arthroplasty showed that transfusion requirements after bilateral simultaneous THR were expected to be higher than after staged or unilateral THR, but there was no difference in the time to surgery between the two groups (18). One possible explanation for this is that blood transfusion practices and reporting may vary between hospitals and surgeons.

Hip replacements are procedures that are mainly associated with older patients, with an average age of 70 years for primary hip replacements in the UK (19). Worldwide, national populations are aging, with declining fertility and mortality rates in most countries. Globally, the number of people over 65 years old is expected to rise to 1.6 billion by 2050 (20). Thus, the number of patients requiring bilateral hip replacements will continue to increase. This study provides important evidence regarding the safety of bilateral hip replacement surgeries, which is of great significance as the popularity of this surgery increases.

A previous study evaluated 11,676 patients who underwent total joint arthroplasty, including 6,604 patients undergoing THRs. Multivariate analysis indicated that increased blood loss was associated with males (21). However, other studies have shown that patient-related factors are associated with a higher risk of receiving a blood transfusion, including female sex (22, 23). A recent study showed that men had an increased risk of multiple individual adverse events, including death, surgical site infection, cardiac arrest, return to the operating room, and readmission. Conversely, women had an increased risk of urinary tract infection and blood transfusion (24). Our study failed to indicate any sex-related differences in perioperative safety that were manifest as significant differences in complication rates, total blood losses, or operative times. Our data included only patients from one hospital and the sample size was small; multi-site studies are more likely to see variances in blood transfusion practices and reporting between hospitals and surgeons, which may result in different results between studies.

Studies have not demonstrated statistical perioperative complication rate differences in patients undergoing TKRs or THRs across BMI categories (25). One retrospective study showed that overweight patients undergoing primary THR have a higher risk of increased surgical time and intraoperative blood loss, particularly if they present with multiple comorbidities (26). A recent study showed that obesity could increase perioperative blood loss but did not increase transfusion risk for patients undergoing simultaneous bilateral THRs. Conversely, obese and overweight patients may have lower transfusion needs than normal-weight patients because of their higher blood volumes. Additionally, obesity was not observed to affect the incidence of complications (27). Similarly, we found that the operative time was shorter for overweight patients in the bilateral group than in the unilateral group; moreover, there were no significant differences in total blood losses or operative times between the unilateral and bilateral groups in the other BMI groups.

In addition, it has been shown that pre- and post-operative C-reactive protein (CRP) levels, erythrocyte sedimentation rate (ESR) levels, and white blood cell (WBC) counts can predict periprosthetic infection and prognosis after hip arthroplasty (28). There were no statistical differences in CRP levels between unilateral and bilateral hip replacement patients in this study. Apart from CRP, there was no statistical difference between the two groups in other blood parameters. It is expected that there should be no difference in the rate of late periprosthetic infection in patients. Specific results need to be supplemented by follow-up.

This study had some limitations. The first is the limited number of patients included in this single-center following-up study. Second, there was a lack of data regarding preoperatively prescribed medications, which may result in an evaluation bias of factors affecting operation outcomes. Moreover, in this study, we haven't operated the examinations of heart and lung function. We will assess these two examinations in the next future. Finally, we did not assess whether there was a difference in the prognostic survival curves between the different groups.

## Conclusion

This study evaluated safety differences among patients undergoing unilateral and bilateral hip replacements. The findings suggest that patients requiring bilateral THRs do not have a higher risk of elevated blood loss or prolonged surgical time if they undergo simultaneous replacement, compared with patients undergoing staged bilateral or unilateral replacements. Thus, simultaneous bilateral THRs are safe and should be considered for candidate patients.

## Data Availability

The raw data supporting the conclusions of this article will be made available by the authors, without undue reservation.
